# Supplementary material for: Noise reduction strategies in metagenomic chromosome confirmation capture to link antibiotic resistance genes to microbial hosts
Source: Microb Genom. 2023 Jun 5;9(6):mgen001030. doi: 10.1099/mgen.0.001030 (PMC10327510; doi:10.1099/mgen.0.001030)
Supplement: Supplementary material 1 [file mgen-9-1030-s001.pdf]

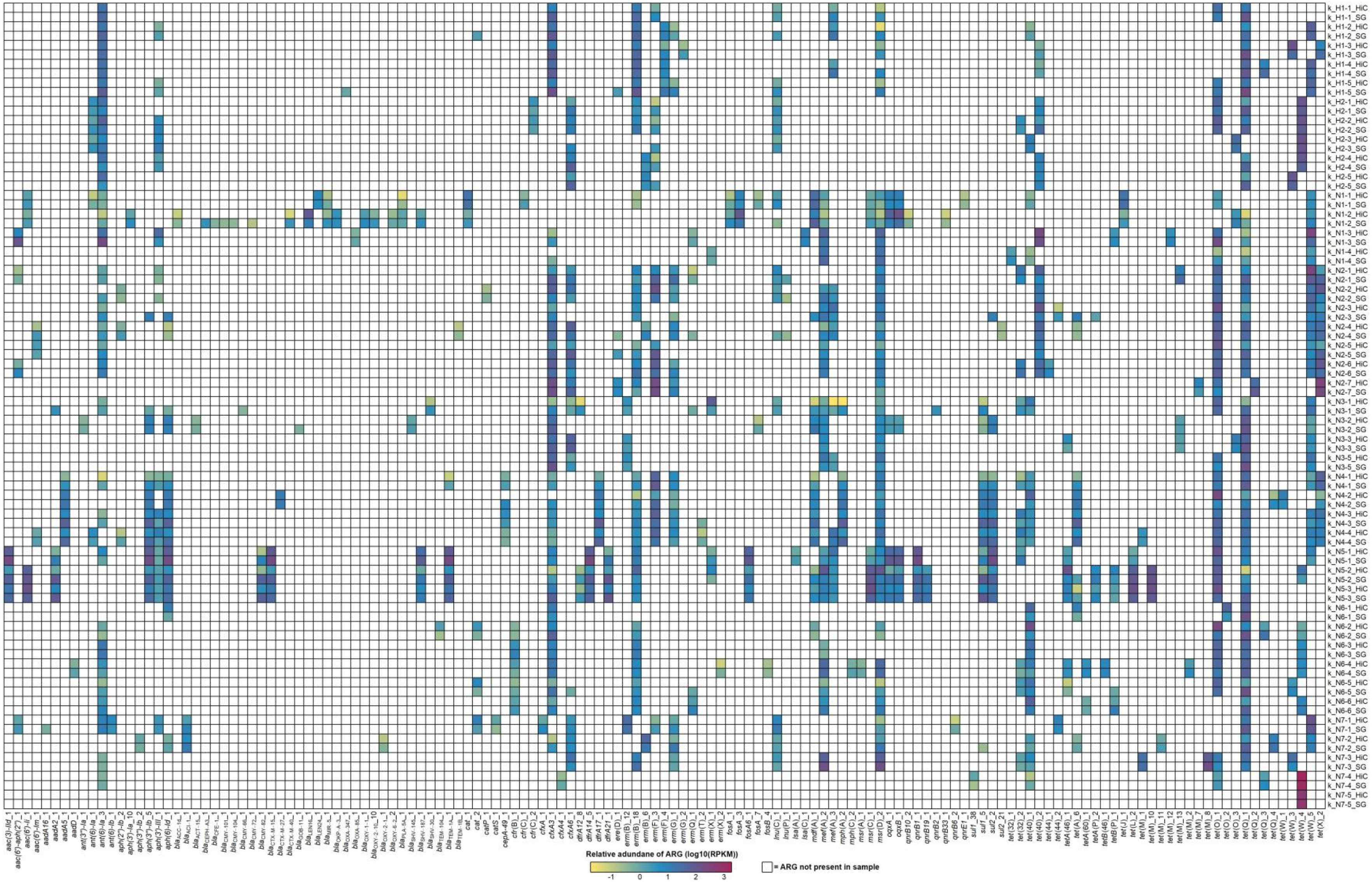

Supplementary Figure 1. ARG abundance in the K_HiC dataset.

ARG sequences from the assemblies were isolated, and the reads from each dataset were mapped to the ARGs (columns). The relative abundance was calculated as reads per kilobase per million mapped reads (RPKM). White cells mean the ARG was not present, and coloured cells show that the ARG was present, with the colour relating to the relative abundance of the ARG within that set of reads (log(10) transformed RPKM values). Different datasets are separated by gaps in the heatmap. The individual datasets show RPKM of the shotgun reads (*_SG) or Hi-C reads (*_HiC) mapping to ARGs identified in the shotgun metagenomic assembly.

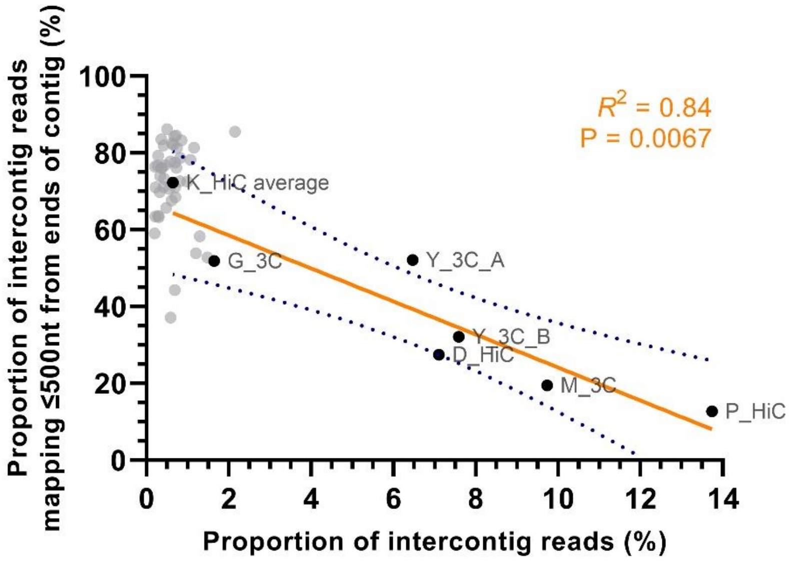

Supplementary Figure 2. The proportion of identified intercontig reads vs the proportion of intercontig reads mapping within the first or last 500 nucleotides (nt) of a contig for all 3C/Hi C datasets.

Each point represents a different 3C/Hi-C sample (labelled). Unlabelled grey points are the individual samples in the K_HiC dataset. Slope calculated via linear regression analysis showing a statistically significant correlation (P = 0.0067, Spearman correlation). Blue dotted lines indicate the 95% confidence interval.

Supplementary Table 1. Read counts during meta3C/Hi-C analysis

Dataset	G_3C	M_3C	Y_3C		D_HiC	P_HiC	K_HiC*
			Y_3C_A	Y_3C_B			
**Processed reads**	198,493,086	366,961,002	2,921,579,828	1,239,950,680	133,509,800	157,755,162	37,984,239
**Reads mapped (MAPQ>20)**	181,467,148	278,726,053	2,868,601,794	1,155,939,113	108,556,752	124,877,330	32,412,518
**Percentage mapped**	91.42%	75.96%	98.19%	93.22%	81.31%	79.16%	85.66%
**Intercontig reads**	3,271,007	35,717,451	188,322,547	94,104,831	9,488,683	21,679,019	192,510
**Percentage intercontig**	1.65%	9.73%	6.45%	7.59%	7.11%	13.74%	0.64%
**Filtered intercontig reads**	1,574,468	28,773,234	90,197,910	63,855,164	6,880,255	18,917,493	53,157
**Percentage intercontig after filtering**	0.79%	7.84%	3.09%	5.15%	5.15%	11.99%	0.18%

*for K_HiC, an average of 43 samples is presented in this table; For datasets that used multiple restriction enzymes, numbers presented are a combined total; MAPQ = mapping quality

Supplementary Table 2. Comparison of spike-in WGS and G_3C reads that map to spike-in genomes

**Spike-in**	***E. coli* E3090**		***E. faecium* E745**	
**Dataset**	**G_3C**	**WGS**	**G_3C**	**WGS**
**Total reads**	14,497,782	1,284,538	8,170,430	3,333,334
**Reads mapped to G_3C assembly (MAPQ>20)**	14,237,338	1,256,272	7,764,542	3,175,581
**%mapped to G_3C assembly**	98.20%	97.80%	95.03%	95.27%
**Intercontig reads**	141,473	10,080	81,191	78,106
**%intercontig reads**	0.98%	0.78%	0.99%	2.34%

WGS = whole genome sequencing; MAPQ = mapping quality; nt = nucleotides
